# Overweight and obesity are associated with cardiac adverse structure remodeling in Chinese elderly with hypertension

**DOI:** 10.1038/s41598-019-54359-9

**Published:** 2019-11-29

**Authors:** Cheng Liu, Gang Li, Jari A. Laukkanen, Lan Hao, Qianping Zhao, Jing Zhang, Xu Zhang

**Affiliations:** 1 Division of Cardiology, Department of Geriatrics and General Practice, The First Affiliated Hospital of Chongqing Medical University, Chongqing, China; 2 Institute of Public Health and Clinical Nutrition, University of Eastern Finland, Kuopio, Finland; 3 Faculty of Sport and Health Sciences, University of Jyväskylä, Jyväskylä, Finland; 4 Chongqing Key Laboratory of Ultrasound Molecular Imaging, Institute of Ultrasound Imaging, Chongqing Medical University, Chongqing, China

**Keywords:** Cardiac device therapy, Obesity

## Abstract

There is limited information on the association of overweight and obesity with cardiac remodeling in elderly population. Therefore, we investigated whether overweight and obesity are associated with cardiac geometric structures and function in Chinese elderly. A total of 1183 hospitalized patients (aged 65–99 years) with primary hypertension were collected retrospectively in a cross-sectional study, and divided into underweight, normal weight, overweight and obesity patient groups according to their body mass index (BMI). Cardiac echocardiographic parameters were compared between the groups. BMI was 17.2 ± 1.2, 21.4 ± 1.2, 25.1 ± 1.2, 30.2 ± 2.6 kg/m^2^ in underweight, normal weight, overweight and obesity groups respectively. Aortic and left atrial diameter, interventricular septal and left ventricular (LV) posterior wall thickness, LV end-diastolic and end-systolic diameter, and indexed LV mass, and prevalence of E/A reversal were higher, while LV ejection fraction and fractional shortening were lower in elderly with overweight or obesity, as compared with whose with underweight or normal weight separately (All *P* < 0.05). However, multivariable regression analysis showed that overweight and obesity are independently related to increased LV wall thickness, end-diastolic diameter and mass (All *P* < 0.05). In conclusions, this study demonstrates that overweight and obesity are associated with increased LV wall thickness, end-diastolic diameter and mass in Asian elderly.

## Introduction

A U-shaped association between body mass index (BMI) and mortality exists in elderly population^1,2^. The mortality rate has been higher in underweight elderly patients than those with normal bodyweight^1–3^. Unexpectedly, a study has shown that the mortality is lower in elderly with overweight or mild moderate obesity than whose with normal bodyweight^4^, although overweight and obese patients usually have comorbidities such as hypertension, type 2 diabetes mellitus and dyslipidemia than whose with normal bodyweight^5,6^. This phenomenon is widely known as obesity paradox^5^. However, unhealthy obesity has been associated with increased all-cause mortality among elderly subjects^5^. The morbid obesity is related to higher prevalence of hypertension, diabetes and dyslipidemia, which could be associated with major adverse cardiovascular consequences in elderly patients. Nevertheless, the study on detailed associations of overweight and obesity with cardiac structures and function is still limited in elderly Asian population. Therefore, we collected data on Chinese elderly patients with primary hypertension in a cross-sectional study in order to investigate the relationship of overweight and obesity to cardiac remodeling and function.

## Methods

### Patients

One thousand one hundred eighty-three hospitalized elderly (aged 65 to 99 years, 538 males, 645 females) with primary hypertension were collected retrospectively from April 2014 to March 2018 in a cross-sectional study. The hypertensive patients with or without comorbidities were treated with antihypertensive drugs or/and lifestyle modification. Among them, 98 subjects were firstly diagnosed with primary hypertension; 103 subjects were treated only with lifestyle modification. Hypertension criteria included an average untreated casual systolic blood pressure (SBP) ≥ 140 mm Hg or diastolic blood pressure (DBP) ≥ 90 mm Hg at clinical visits^7,8^. Type 2 diabetes mellitus (DM) was defined by fasting plasma glucose (FPG) ≥ 7 mmol/L or 2 hours postprandial plasma glucose (PPG) ≥ 11.1 mmol/L in oral glucose tolerance test; Moreover, plasma insulin concentration is normal or increased in the subjects^9^. Fatty liver was diagnosed by color doppler ultrasound. Gout was considered when there were arthralgia, arthritis and hyperuricemia or gouty tophus. Coronary heart disease (CHD) was considered when there was a history of coronary revascularization, coronary angiographic or electrocardiographic evidences of myocardial infarction or ischemia accompanied by anti-anginal medications. Stroke was based on the history and computer tomographic or magnetic resonance imaging relevant evidences. Chronic obstructive pulmonary disease (COPD) was diagnosed by a history of chronic cough with sputum and pulmonary obstructive ventilation dysfunction. Data was collected retrospectively from the First Affiliated Hospital of Chongqing Medical University, Chongqing, China. The study was approved by the ethics committee of the institution. However, the institutional ethics committee waived the need for patients’ written informed consent for this retrospective analysis of clinically acquired data.

We excluded patients with secondary hypertension due to glomerulonephritis, pyelonephritis, aldosteronism, vascular diseases and glucocorticoid use. The patients with primary hypertension coexisting senile degenerative cardiac valvular disease, congenital heart disease or whose treated with bariatric surgery were also excluded.

### Assessment of body mass index

BMI = body weight kg/(height m)^2^. Underweight, normal weight, overweight and obesity were respectively diagnosed according to World Health Organization criteria for Asian populations, as considering that overweight and obese Asian people are more susceptible to cardiovascular diseases^10^. Underweight refers BMI < 18.5 kg/m^2^. Normal weight: 23 kg/m^2^ < BMI ≥ 18.5 kg/m^2^. Overweight: 27.5 kg/m^2^ < BMI ≥ 23 kg/m^2^. Obesity: BMI ≥ 27.5 kg/m^2^^10^.

### Measurement of blood glucose, lipids, uric acid, creatinine and eGFR

Plasma glucose and glycated hemoglobin (HbA1c) concentrations were determined by hexokinase method and high performance liquid ion exchange chromatography respectively. Total cholesterol (TC), high density lipoprotein cholesterol (HDL-C) and triglyceride (TG) were determined by cholesterol oxidase, magnesium sulfate precipitation and three-color methods respectively. Low density lipoprotein cholesterol (LDL-C) was calculated by TC - HDL-C - (TG/2.19). Plasma uric acid and creatinine concentrations were determined by uricase and enzyme methods respectively. eGFR (estimated glomerular filtration rate) was calculated by following equations. For male, serum creatinine (Scr) > 80 μmol/L: eGFR = 141 · (Scr μmol/L/88.4/0.9)^−1.209^ · 0.993^Age years^; Scr ≤ 80 μmol/L: eGFR = 141 · (Scr μmol/L/88.4/0.9)^−0.411^ · 0.993^Age years^. For female, Scr > 62 μmol/L: eGFR = 144 · (Scr μmol/L/88.4/0.7)^−1.209^ · 0.993^Age years^; Scr ≤ 62 μmol/L: eGFR = 144 · (Scr μmol/L/88.4/0.7)^−0.329^ · 0.993^Age years^^11^_._

### Measurement of cardiac structure and function by echocardiography

According to American Society of Echocardiology leading-edge method, transthoracic color doppler echocardiographic studies were performed and read by experienced cardiologists in a single core laboratory in our hospital, with a Vivid echocardiographic system (General Electric Co., Fairfield, CT, USA), which was equipped with a 3.5-MHz sector scan transducer^12,13^. Two-dimensional–guided M-mode measurement of right ventricular diameter (RVD), aortic diameter (AOD), interventricular septal thickness (IVST), left ventricular (LV) posterior wall thickness (LVPWT) and end-diastolic diameter (LVEDD) were examined at end-diastole from the parasternal long-axis view; Left atrial diameter (LAD) and LV end-systolic diameter (LVESD) were measured at end-systole from the parasternal long-axis view; LV mass (LVM) g = (0.80 · 1.04 · ((LVEDD mm + IVST mm + LVPWT mm)^3^ - (LVEDD mm)^3^) + 0.6) · 0.001; LVMI g/m^2.7^ = LVM g/(height m)^2.7^; LV peak early (E) and late (A) filling velocities were measured. E/A reversal prevalence, LV ejection fraction (LVEF) and fractional shortening (LVFS) were examined^8,12,13^.

### Statistical analysis

Continuous parameters were expressed as mean values ± standard deviation (SD). The statistical differences were evaluated by one-way ANOVA, followed, in case of significance, by a two-side LSD test for multiple comparisons including between overweight and underweight or normal weight groups, and between obesity and underweight or normal weight groups. Categorical data were summarized as percentages and compared using Chi-square test. Pearson univariate linear correlation and logistic multivariate regression models were utilized for data analysis. Statistical analysis was performed by SPSS 22.0 software package (IBM Company, Chicago, Illinois 60606, USA). A 2-tailed value of *P* < 0.05 was considered to be statistically significant.

## Results

### Clinical characteristics

The study included 1183 hypertensive patients with mean age and BMI (SD) of 79 ± 7 years and 24.5 ± 3.8 kg/m^2^ respectively. The male sex, BMI, prevalence of hypertension, dyslipidemia and type 2 DM, plasma glucose, HbA1c, fatty liver markers, uric acid, cigarette smoking, alcohol drinking, and the use of medications were higher in overweight patients as compared with underweight or normal weight patients (All *P* < 0.05, Tables 1–2). Consistently, the above mentioned parameters have increased in the obese patients as compared with the underweight or normal weight patients, as shown in the Tables 1–2. BMI was correlated positively with male sex, hypertension, type 2 DM, plasma glucose, HbA1c, dyslipidemia, fatty liver, gout, uric acid, cardiac functional class, cigarette smoking, alcohol drinking, and use of medications respectively (All *P* < 0.05, Tables 1–2).

**Table 1:** Comparison of clinical parameters among underweight, normal weight, overweight and obesity groups, and univariate correlations of the parameters with BMI

	Underweight n = 61	Normal weight n = 375	Overweight n = 530	Obesity n = 217	r	*P* value
Sex, M/F, n	19/42	146/229	269/261*^†^	104/113*^†^	0.073	0.012
Age, year	82.3 ± 7.0	79.3 ± 7.5*	78.9 ± 7.2*	78.5 ± 7.4*	−0.089	0.002
BMI, kg/m^2^	17.2 ± 1.2	21.4 ± 1.2*	25.1 ± 1.2*^†^	30.2 ± 2.6*^†‡^	1.000	<0.001
Body weight, kg	42.6 ± 6.4	53.8 ± 6.8*	64.4 ± 7.6*^†^	75.5 ± 9.2*^†‡^	0.799	<0.001
Height, m	1.6 ± 0.1	1.6 ± 0.1	1.6 ± 0.1	1.6 ± 0.1	−0.017	0.560
Smoking, n (%)	10 (16.4)	81 (21.6)*	153 (28.9)*^†^	62 (28.6)*^†^	0.085	0.003
Drinking, n (%)	1 (1.6)	48 (12.8)*	84 (15.8)*	39 (18.0)*^†^	0.087	0.003
HT duration, year	10.8 ± 10.7	14.5 ± 12.8*	14.6 ± 12.2*	16.4 ± 12.6*	0.086	0.003
HT stage	2.6 ± 0.6	2.6 ± 0.6	2.6 ± 0.6	2.6 ± 0.6	0.025	0.386
SBP, mmHg	140.5 ± 26.3	142.4 ± 21.7*	143.7 ± 20.4*	143.7 ± 20.6*	0.088	0.003
DBP, mmHg	74.4 ± 12.3	75.2 ± 13.1	75.4 ± 13.1	74.7 ± 11.7	0.030	0.333
Type 2 DM, n (%)	11 (18.0)	139 (37.1)*	213 (40.2)*	105 (48.4)*^†‡^	0.129	<0.001
DM duration, year	1.8 ± 4.7	3.5 ± 6.4*	4.2 ± 7.5*	4.5 ± 6.5*	0.076	0.009
Fatty liver, n (%)	1 (1.6)	23 (6.1)*	119 (22.5)*^†^	85 (39.2)*^†‡^	0.312	<0.001
Gout, n (%)	1 (1.6)	9 (2.4)	23 (4.3)	17 (7.8)*^†^	0.086	0.003
CHD, n (%)	22 (36.1)	111 (29.6)	199 (37.5)	83 (38.2)	0.052	0.054
Resting HR, beats/min	70.8 ± 11.6	71.3 ± 12.2	69.7 ± 10.1	70.9 ± 10.0	−0.005	0.863
Class (NYHA)	1.5 ± 0.8	1.4 ± 0.8	1.5 ± 0.8	1.7 ± 0.9^†^	0.088	0.003
Atrial fibrillation, n (%)	13 (21.3)	41 (10.9)	57 (10.8)	28 (12.9)	−0.016	0.580
Stroke, n (%)	28 (45.9)	179 (47.7)	236 (44.5)	82 (37.8)	−0.056	0.054
COPD, n (%)	13 (21.3)	40 (10.7)	62 (11.7)	23 (10.6)	−0.050	0.088
FBG, mmol/L	5.8 ± 2.1	6.1 ± 2.2	6.3 ± 2.0*	6.8 ± 2.4*^†‡^	0.113	<0.001
HbA1c, %	6.0 ± 0.70	6.2 ± 1.2	6.5 ± 1.2*	6.7 ± 1.2*^†^	0.151	<0.001
TG, mmol/L	1.2 ± 1.1	1.4 ± 1.4*	1.5 ± 1.3*^†^	1.6 ± 1.0*^†^	0.075	0.012
TC, mmol/L	4.0 ± 1.0	4.1 ± 1.1	4.0 ± 1.0	3.9 ± 1.0	−0.044	0.140
LDL-C, mmol/L	2.1 ± 0.8	2.4 ± 0.9	2.4 ± 0.9	2.3 ± 0.9	0.025	0.386
HDL-C, mmol/L	1.5 ± 0.5	1.4 ± 0.4	1.2 ± 0.6*^†^	1.2 ± 0.3*^†^	−0.214	<0.001
Uric acid, umol/L	287.1 ± 104.2	321.5 ± 106.2*	351.6 ± 101.5*^†^	361.4 ± 93.7*^†^	0.182	<0.001
Creatinine, umol/L	93.9 ± 60.1	86.0 ± 39.0	92.3 ± 51.8	90.6 ± 37.6	0.018	0.539
eGFR, ml/min/1.73 m^2^	65.4 ± 25.4	68.5 ± 20.1	66.9 ± 19.6	66.5 ± 19.4	−0.021	0.471

Values presented as mean ± SD or n (%); *p < 0.05 versus Underweight group; ^**†**^*p* < 0.05 versus Normal weight group; ^**‡**^*p* < 0.05 versus Overweight group. BMI, body mass index; HT, hypertension; SBP, systolic blood pressure; DBP, diastolic blood pressure; DM, diabetes mellitus; CHD, coronary heart disease; HR, heart rate; NYHA, New York heart association cardiac function classfication; COPD, chronic obstructive pulmonary disease; FPG, fast plasma glucose; HbA1c, glycated hemoglobin; TG, triglyceride; TC, total cholesterol; LDL-C, low density lipoprotein cholesterol; HDL-C, high density lipoprotein cholesterol; eGFR, estimated glomerular filtration rate.

**Table 2:** Comparison of medication use among underweight, normal weight, overweight and obesity groups, and univariate correlations of the parameters with BMI

	Underweight n = 61	Normal weight n = 375	Overweight n = 530	Obesity n = 217	r	*P* value
ACEI, n (%)	9 (14.8)	38 (10.1)	47 (8.9)	25 (11.5)	−0.006	0.847
ARB, n (%)	21 (34.4)	197 (52.5)*	339 (64.0)*^†^	149 (68.7)*^†^	0.167	<0.001
β1 blocker, n (%)	26 (42.6)	116 (30.9)	159 (30.0)	72 (33.2)	−0.003	0.914
CCB, n (%)	28 (45.9)	177 (47.2)	301 (56.8)*^†^	128 (59.0)*^†^	0.091	0.002
Furosemide, n (%)	8 (13.1)	25 (6.7)	52 (9.8)	20 (9.2)	0.032	0.276
Thiazides, n (%)	3 (4.9)	16 (4.3)	41 (7.7)	18 (8.3)	0.042	0.143
Spirolactone, n (%)	8 (13.1)	25 (6.7)	51 (9.6)	18 (8.3)	0.003	0.931
Insulin, n (%)	4 (6.6)	46 (12.3)*	84 (15.8)*	42 (19.4)*^†^	0.108	<0.001
Metformin, n (%)	0 (0.0)	42 (11.2)*	86 (16.2)*^†^	56 (25.8)*^†‡^	0.173	<0.001
Acarbose, n (%)	6 (9.8)	61 (16.3)	98 (18.5)	42 (19.4)	0.043	0.141
Sulfonylureas, n (%)	3 (4.9)	18 (4.8)	30 (5.7)	17 (7.8)	0.044	0.135
Statins, n (%)	42 (68.9)	305 (81.3)*	445 (84.0)*	182 (83.9)*	0.066	0.023
Fibrates, n (%)	1 (1.6)	2 (0.5)	6 (1.1)	1 (0.5)	−0.015	0.614
Bicarbonate, n (%)	0 (0.0)	9 (2.4)*	18 (3.4)*	12 (5.5)*	0.058	0.045
Nitrates, n (%)	23 (37.7)	102 (27.2)	160 (30.2)	63 (29.0)	−0.008	0.795
Trimetazidine, n (%)	23 (37.7)	97 (25.9)	158 (29.8)	66 (30.4)	−0.002	0.938
Asprin, n (%)	17 (27.9)	133 (35.5)*	218 (41.1)*^†^	87 (40.1)*^†^	0.065	0.025
Clopidogrel, n (%)	25 (41.0)	171 (45.6)	215 (40.6)	81 (37.3)	−0.055	0.056
Warfarin, n (%)	5 (8.2)	17 (4.5)	33 (6.2)	14 (6.5)	0.011	0.697
Digoxin, n (%)	2 (3.3)	5 (1.3)	10 (1.9)	2 (0.9)	−0.041	0.155

Values presented as mean ± SD or n (%); ^*^p < 0.05 versus Underweight group; ^**†**^*p* < 0.05 versus Normal weight group; ^**‡**^*p* < 0.05 versus Overweight group. BMI, body mass index; ACEI, angiotensin converting enzyme inhibitor; ARB, angiotensin II receptor inhibitor; CCB, calcium channel blocker.

### Cardiac LV structure and function

Echocardiographic parameters were also compared between overweight and underweight or normal weight patients. The results showed that AOD, LV wall thickness, diameter and mass, and the prevalence of E/A reversal were increased; whereas LVEF and LVFS were decreased in patients with overweight (All *P* < 0.05, Table 3). The respective comparisons between obese and underweight or normal weight patients showed that AOD, LAD, LV wall thickness, diameter and mass, and the prevalence of E/A reversal were increased; but LV function was decreased in patients with obesity (All *P* < 0.05, Table 3). BMI was directly related to echocardiographic parameters including AOD, LAD, LV wall thickness, diameter, mass and the prevalence of E/A reversal; Meanwhile it was inversely related to LVEF and LVFS (All *P* < 0.05, Table 3).

**Table 3:** Comparison of echocardiographic parameters among underweight, normal weight, overweight and obesity groups, and univariate correlations of the parameters with BMI

	Underweight n = 61	Normal weight n = 375	Overweight n = 530	Obesity n = 217	r	*P* value
RVD, mm	18.3 ± 2.8	18.6 ± 2.0	18.7 ± 1.5	19.0 ± 1.6	0.051	0.056
AOD, mm	27.6 ± 2.6	28.3 ± 2.9*	28.7 ± 2.6*^†^	29.3 ± 2.9*^†‡^	0.166	<0.001
LAD, mm	29.9 ± 6.8	29.7 ± 5.4	30.4 ± 4.6	31.5 ± 4.8*^†‡^	0.140	<0.001
IVST, mm	10.9 ± 1.6	11.0 ± 1.4	11.3 ± 1.2*^†^	11.6 ± 1.3*^†‡^	0.175	<0.001
LVPWT, mm	10.8 ± 1.3	10.9 ± 1.3	11.2 ± 1.2*^†^	11.5 ± 1.2*^†‡^	0.194	<0.001
LVDD, mm	44.4 ± 4.5	45.8 ± 4.2*	47.2 ± 4.6*^†^	47.9 ± 4.6*^†^	0.212	<0.001
LVSD, mm	29.3 ± 3.5	30.3 ± 3.4*	31.6 ± 4.2*^†^	32.0 ± 3.9*^†^	0.192	<0.001
LVM, g	170.6 ± 45.0	181.0 ± 45.5	197.6 ± 47.7*^†^	209.0 ± 46.2*^†‡^	0.243	<0.001
LVMI, g/m^2.7^	50.5 ± 13.1	52.6 ± 13.2	55.8 ± 13.0*^†^	60.8 ± 12.8*^†‡^	0.264	<0.001
LVFS, %	34.2 ± 3.0	33.9 ± 3.1	33.4 ± 3.4*^†^	33.4 ± 3.0*^†^	−0.072	0.014
LVEF, %	63.4 ± 3.5	62.7 ± 4.5	61.7 ± 5.1*^†^	61.8 ± 4.5*^†^	−0.091	0.002
E/A < 1, n (%)	37 (60.7)	270 (72.0)*	407 (76.8)*	167 (77.0)*	0.071	0.015

Values presented as mean ± SD or n (%); ^*^p < 0.05 versus Underweight; ^**†**^*p* < 0.05 versus Normal weight group; ^**‡**^*p* < 0.05 versus Overweight group. BMI, body mass index; RVD, right ventricular diameter; AOD, aortic diameter; LAD, left atrial diameter; IVST, interventricular septal thickness; LVPWT, left ventricular posterior wall thickness; LVEDD, left ventricular end-diastolic diameter; LVESD, left ventricular end-systolic diameter; LVM, left ventricular mass; LVMI, left ventricular mass index; LVFS, left ventricular fractional shortening; LVEF, left ventricular ejection fraction; E/A: left ventricular peak early (E) and late (A) filling velocity ratio.

Multivariable adjusted regression analysis showed that BMI was not related to sex, age, cigarette smoking, alcohol drinking, hypertension, diabetes, dyslipidemia, fatty live and gout (All *P* > 0.05, Table 4). However, BMI was directly associated only with LV wall thickness, end-diastolic diameter and mass (All *P* < 0.05, Table 4).

**Table 4:** Multivariate regression analysis of parameters associated with BMI

	β	SE	Wald	*P* value	95% Confidence
SBP	0.000	0.011	0.000	0.984	−0.022–0.022
Type 2 DM	0.509	0.629	0.655	0.418	−0.724–1.741
TG	0.215	0.201	1.140	0.286	−0.180–0.609
Gout	−1.032	1.206	0.732	0.392	−3.396–1.332
AOD	−0.030	0.099	0.093	0.760	−0.225–0.165
LAD	−0.106	0.075	1.955	0.162	−0.253–0.042
IVST	−1.824	0.746	5.975	0.015	−3.286–(−0.361)
LVPWT	−3.013	0.789	14.587	<0.001	−4.560–(−1.467)
LVDD	−1.067	0.428	6.211	0.013	−1.907–(−0.228)
LVSD	−0.247	0.365	0.456	0.500	−0.963–0.469
LVM	−0.182	0.054	11.276	0.001	−0.287–(−0.076)
LVMI	1.248	0.165	57.574	<0.001	0.926–1.571
LVFS	0.129	0.214	0.365	0.546	−0.291–0.550
LVEF	−0.241	0.162	2.204	0.138	−0.560–0.077
E/A < 1	0.390	0.781	0.250	0.617	−1.139–1.920

BMI, body mass index; SBP, systolic blood pressure; DM, diabetes mellitus; TG, triglyceride; AOD, aortic diameter; LAD, left atrial diameter; IVST, interventricular septal thickness; LVPWT, left ventricular posterior wall thickness; LVEDD, left ventricular end-diastolic diameter; LVESD, left ventricular end-systolic diameter; LVM, left ventricular mass; LVMI, left ventricular mass index; LVFS, left ventricular fractional shortening; LVEF, left ventricular ejection fraction; E/A: left ventricular peak early (E) and late (A) filling velocity ratio.

## Discussion

The present study shows that overweight and obesity were associated with increased LV wall thickness, dimension and mass in the hypertensive elderly Asian population. The results remained consistent, after adjustment of many variables including sex, age, hypertension, diabetes, dyslipidemia, gout, use of cigarette smoking and alcohol drinking.

Overweight and obesity may be the most important lifestyle-related factors involving in cardiovascular morbidity and mortality in elderly^14–16^. Previous studies showed that overweight and obese older people are susceptible to cardiovascular diseases in Asian populations^14,15^. Our study showed that even slightly increased BMI was associated with unwanted cardiac remodeling. On the other hand, it has suggested that overweight and obesity with cardiac remodeling could be prevented through beneficial lifestyle modification and bariatric surgery^17,18^.

Through its linking with the occurrence of other metabolic syndrome components, obesity is closely related to atherosclerotic cardiovascular disease and consequent hemodynamic alteration as well as cardiac structural change and dysfunction^19^. The pathophysiological mechanisms underlying the overweight- and obesity- LV remodeling may be multifactorial. With increments in adipose tissue and its vascular bed size in the case of overweight or obesity, as a compensatory mechanism, the sympathetic and renin-angiotensin-aldosterone (RAA) systems are activated to drive cardiovascular system overload for meeting the metabolic demands of extra adipose tissue. Long-standing overweight leads to cardiac volume- and pressure-overload, and higher cardiac output and blood pressure via activation of sympathetic and RAA systems, consequently causing myocardial fibrosis, ventricular maladaptive hypertrophy and enlargement with cardiac dysfunction, and increase of cardiovascular events and mortality^20^.

Excessive fat from adipose tissues accumulates in vessels, leading to arterial stiffness and hypertension^21^; as adipose accumulates in respiratory tract, it mechanically impairs pulmonary ventilation. Resultantly, hypoventilation, obstructive sleep apnea, hypoxia, hypercapnia and respiratory acidosis can then occur^22^. This chronic hypoxia and hypercapnia cause sympathetic-RAA system activation, vasoconstriction and hypertension with consequent overweight- or obesity-related cardiac geometric remodeling and dysfunction^20,22^. Additionally, accumulated adipose tissue can secrete RAA components, which can activate sympathetic and RAA systems, leading to adverse cardiac dysfunction^23,24^.

The present study has demonstrated that both overweight and obesity are directly related to LV wall thickness, LV dilation and mass in elderly^25^. Our results are in line with other studies, which have confirmed that overweight and obesity predominantly resulted in LV concentric hypertrophy^25,26^. Besides these findings, our study also replenished that overweight and obesity are independently related to LV enlargement and larger LV mass in elderly. Although the overweight- and obesity-associated LV enlargement has not been consistently related to LV systolic dysfunction^26^, it may lead to cardiac remodeling and LV diastolic dysfunction^27,28^. Previous epidemiological evidence has also indicated that higher body weight could provide protection against adverse cardiac events in elderly population, which is reflecting so called as obesity paradox^27,29^. However, obesity with significant cardiac remodeling has been associated with an increased risk of cardiac death^16,20^.

Nevertheless, our study suggested that overweight and obesity are not associated with dilation of right ventricle and left atrium and LV function. The underlying causes could be explained as follows: LV geometric alterations were mild and may not cause dilation of right ventricle and left atrium and LV dysfunction^30^. Left atrial dimension was evaluated by anteroposterior diameter, although left atrial volume has been used as a measure of left atrial size. During previous years, left atrial volume was not routinely assessed in clinical practice, and therefore this data was not available from our medical records. Secondly, LV diastolic dysfunction was defined by E/A reversal^31^, which may not precisely reflect LV diastolic dysfunction.

The major limitation of the present study is that the data was obtained from a cross-sectional study. Although multivariable adjusted regression analyses were performed, the effects of other possible confounding factors should be taken into consideration. Therefore, large prospective studies would be needed to reconfirm the observed findings. The study was carried on hypertensive elderly, its results should be cautiously extrapolated to normotensive elderly.

In conclusion, overweight and obesity are related to echocardiographic findings such as increased LV wall thickness, dilation and mass in elderly. These LV changes can be detected easily by cardiac echocardiography in real life clinical practice.
